# The hippocampal extracellular matrix regulates pain and memory after injury

**DOI:** 10.1038/s41380-018-0209-z

**Published:** 2018-09-26

**Authors:** Maral Tajerian, Victor Hung, Huy Nguyen, Gail Lee, Lydia-Marie Joubert, Andrey Victorovich Malkovskiy, Bende Zou, Simon Xie, Ting-Ting Huang, J. David Clark

**Affiliations:** 1 Department of Anesthesiology, Stanford University School of Medicine, Stanford, CA USA; 2 Veterans Affairs Palo Alto Health Care System, Palo Alto, CA USA; 3 Palo Alto Veterans Institute for Research, Palo Alto, CA USA; 4 Department of Neurology and Neurological Sciences, Stanford University, Stanford, CA USA; 5 Department of Biology, Stanford University, Stanford, CA USA; 6 Cell Sciences Imaging Facility, Stanford University, Stanford, CA USA; 7 Department of Biomaterials and Advanced Drug Delivery, Stanford University, Stanford, CA USA; 8 AfaSci Research Laboratories, Mountainview, CA USA

## Abstract

Chronic pain poses a heavy burden for the individual and society, comprising personal suffering, comorbid psychiatric symptoms, cognitive decline, and disability. Treatment options are poor due in large part to pain centralization, where an initial injury can result in lasting CNS maladaptations. Hippocampal cellular plasticity in chronic pain has become a focus of study due to its roles in cognition, memory, and the experience of pain itself. However, the extracellular alterations that parallel and facilitate changes in hippocampal function have not been addressed to date. Here we show structural and biochemical plasticity in the hippocampal extracellular matrix (ECM) that is linked to behavioral, cellular, and synaptic changes in a mouse model of chronic pain. Specifically, we report deficits in working location memory that are associated with decreased hippocampal dendritic complexity, altered ECM microarchitecture, decreased ECM rigidity, and changes in the levels of key ECM components and enzymes, including increased levels of MMP8. We also report aberrations in long-term potentiation (LTP) and a loss of inhibitory interneuron perineuronal ECM nets, potentially accounting for the aberrations in LTP. Finally, we demonstrate that MMP8 is upregulated after injury and that its genetic downregulation normalizes the behavioral, electrophysiological, and extracellular alterations. By linking specific extracellular changes to the chronic pain phenotype, we provide a novel mechanistic understanding of pain centralization that provides new targets for the treatment of chronic pain.

## Introduction

Chronic pain (CP) is characterized by changes in nociception, affect, and cognition [1, 2] and is often resistant to classical treatment partly due to comorbid maladaptive plastic changes in the central nervous system (CNS) [3–7]. Specifically, a role for the hippocampus has been proposed owing to its role in cognition and memory as well as in modulating the overall pain experience. Functional, anatomical, and biochemical changes in the hippocampi of CP patients [8–10] and animal pain models [7, 11] have been reported. Despite interest in the role of hippocampal remodeling in CP, experimental efforts thus far have been focused on cellular mechanisms of plasticity rather than the extracellular environment in which these cells function. This is likely due to both the neuro-centric nature of pain research and the technical difficulties of studying extracellular components. Here we show structural and biochemical alterations in the hippocampal extracellular matrix (ECM) that are linked to pain, cognitive dysfunction, and cellular plasticity in a mouse model of CP. We report a constellation of deficits in working and location memory that are associated with decreased dendritic complexity, altered ECM microarchitecture, decreased ECM rigidity, and dysregulated ECM remodeling. We also report aberrant hippocampal long-term potentiation (LTP) and a reduction in specialized ECM nets around inhibitory interneurons, potentially accounting for the increased LTP. Finally, we show an amelioration of these maladaptive behavioral and physiological/biochemical phenotypes following an intervention to normalize ECM imbalance. These results delineate extracellular mechanisms of pain-related brain plasticity, thereby offering new therapeutic targets that could modulate already established CNS alterations present in CP.

## Materials and methods

### Animals

Male C57BL/6J mice aged 12–14 weeks were purchased from a commercial supplier (Jackson Labs, Sacramento, CA, USA) and were allowed to habituate to the animal facility for a minimum of 10 days prior to the experiments. Mice were housed in groups of 4 on a 12-h light/dark cycle and an ambient temperature of 22 ± 3 °C, with food and water available *ad libitum*. All animal procedures and experimental designs were approved by the Veterans Affairs Palo Alto Health Care System Institutional Animal Care and Use Committee (Palo Alto, CA, USA) and followed the “animal subjects” guidelines of the International Association for the Study of Pain.

### Tibia fracture and cast immobilization

Following the random allocation to the control or the fracture/cast group, mice were anesthetized with 1.5% isoflurane and underwent a distal tibial fracture in the right leg. Briefly, a hemostat was used to make a closed fracture of the right tibia just distal to the middle of the tibia and the hindlimb was wrapped in casting tape (cat. #82001, Scotchcast^TM^ Plus, 3M, UK), as previously described [12]. After the procedure, the mice were given subcutaneous buprenorphine (0.05 mg/kg]) and enrofloxacin (5 mg/kg) for the next 2 days as well as normal saline (1.5 ml, once) for postoperative analgesia, prevention of infection, and prevention of dehydration. Mice were inspected daily to ensure that the cast was positioned properly through the 3-week period of cast immobilization. Mice were provided with chow pellets postoperatively ad libitum; dietary gels were also made available on the cage floor for mice having undergone surgery. Casts were removed 3 weeks after surgery under brief isoflurane anesthesia. Naive, rather than sham (cast only), animals were used as controls since casting alone results in an intermediate phenotype where transient allodynia is observed both in rodents [13] and in humans [14].

### Behavioral testing

All analysis was blinded to the identity and experimental condition of the animal. Mice were habituated to handling by the experimenter for a few minutes each day for 7 days before initiation of the behavioral tests.

#### Mechanical sensitivity

Calibrated monofilaments (Stoelting Co., USA) were applied to the plantar surface of the hind paw and the 50% threshold to withdraw (g) was calculated as previously described [15]. The stimulus intensity ranged from 0.004 to 1.7 g, corresponding to filament numbers 1.65, 2.36, 2.44, 2.83, 3.22, 3.61, 3.84, 4.08, 4.17, and 4.31. For each animal, the actual filaments used within the aforementioned series were determined based on the lowest filament to evoke a positive response (response = flexion reflex) followed by five consecutive stimulations using the up–down method. The filament range and average interval were then incorporated along with the response pattern into each individual threshold calculation.

#### Location memory test

The arena was a square box 45 × 45 × 45 cm^3^ (*L* × *W* × *H*) made of white polyvinyl chloride. Four metallic enclosures (dimensions) were placed at each of the arena corners. Mice were placed into the arena from the middle of the south wall, with the north wall of the arena having a large visual cue (28 × 21.5 cm^2^ [*W* × *H*] sheet with alternating black and white columns [column width = 1.5 cm]). Mice underwent one 10-min experience trial and a recall trial 24 h later. During the experience trial, one of the four metallic enclosures contained a female mouse (unfamiliar to the male mice). The female mouse was absent during the recall trial. Two identical sets of arenas were used for the two trials to ensure the absence of female odors during the recall trial. The total time spent investigating each of the four enclosures was recorded for the experience and recall trials. The percentage of time spent interacting with the enclosure with the female mouse was used as a measure of female interest (day 1) and female location memory (day 2).

#### Y maze

Y maze spontaneous alternation was used to assess rodents’ tendency to explore new environments; they tend to explore a new arm of the maze rather than revisiting a previously entered arm. The Y-shaped testing arena was made of dark blue acrylics and consisted of 3 symmetrical arms (arms A, B, and C) at 120° angle with a dimension of 20 × 8 × 16 cm^3^ (*L* × *W* × *H*) for each arm, each of which was decorated with different black and white visual cues at the end. Each mouse was introduced to the center of the maze and allowed to freely explore the three arms for 10 min. Arm entries—scored when the animal reached the visual cue at the end of each arm—were recorded during the first 5 min of the test. Mice with good working spatial memory were expected to enter a new arm of the maze without immediate reentry to a previously visited arm. A unique triad combination of consecutive arm entries, i.e., ABC, BCA, BAC, ACB, CBA, was used as a measurement of the spontaneous alteration. The percentage of alteration was calculated as (number of unique triad combination)/(total number arm entries−2).

### Dendritic labeling and analysis

All analysis was blinded to the identity and experimental condition of the animal/tissue.

Mice received a single intracranial injection of adeno-associated virus (AAV) DJ-hSyn1 mCherry into the left hemisphere (to label neurons with red fluorescence protein) 7 weeks after injury according to a previously published protocol [16]. The injection coordinates were AP = −2 mm from bregma and ML = 1.6 mm from midline to deliver AAV to the dentate gyrus (DG). The injection depth was 2.2 mm from the dura. A total of 0.5 µl were injected at the rate of 0.1 µl/min.

Animals were sacrificed 72 h after AAV injections. Tissue preparation was carried out as described in “Immunohistochemistry and histology.” Two images per animal were randomly chosen for analysis. All neurons in the selected images were numbered and identified using the “Mark and Count” tool in Image J (Bethesda, USA). Then 10 IDs were randomly selected for further analysis in each *z*-stack image. Soma area was quantified using the Image J software. Dendritic length, number, and arborization were measured using manual tracing in Neuron J (Bethesda, USA). Sholl analysis was carried out using Fiji (Bethesda, USA) with the Sholl radius set at 5 μm.

### Scanning electron microscopy (SEM) and atomic force microscopy (AFM)

All analysis was blinded to the identity and experimental condition of the animal/tissue. Following ketamine/xylazine anesthesia (300 µl/mouse) and transcardiac perfusion with 1× phosphate-buffered saline (PBS), hippocampi were dissected and decellularized per previously published methods [17]. Decellularization was validated by the absence of DAPI (4,6-diamidino-2-phenylindole; nuclear marker) and OsO_4_ (lipid membrane marker) staining in samples chosen at random.

#### Scanning electron microscopy

Decellularized brain slices (2 mm thick) were fixed in 4% paraformaldehyde with 2% glutaraldehyde in 0.1 M sodium cacodylate buffer (pH7.4) for 24 h at 4 °C. Samples were then briefly rinsed in the same buffer before post-staining with 1% OsO_4_ for 1 h. OsO_4_-treated samples were rinsed in water and gradually dehydrated in increasing concentrations of ethanol (50, 70, 90 100, 100%, 10 min each). Samples were then stacked horizontally onto wire mesh dividers to keep them flattened and critically point dried with liquid CO_2_ using a Tousimis Autosamdri 815A apparatus and 10 min purge time (Tousimis, USA). Dried samples were mounted onto Aluminum SEM stubs using conductive copper tape and sputter-coated (20 Å, Au/Pd) before imaging with a Zeiss Sigma FESEM using InLens SE detection at 3 kV operating voltage (Carl Zeiss Microscopy Inc, USA). ECM abundance and fiber diameter were analyzed using Image J (Bethesda, USA) and microarchitectural motifs were analyzed using Mountainsmap (Digital Surf, France).

#### Atomic force microscopy

AFM force–distance measurements were carried out using a Park Systems NX-10 AFM in a liquid cell with deionized water. Tip sensitivity calibration was performed on glass surface after the experiments. The tip used for the experiments (NanoAndMore, *k* = 0.08 N/m, *R* = 1000 nm) had a SiO_2_ sphere on its apex. The stiffness of tips from this batch was confirmed by the Sader method and not found to differ significantly from the nominal reported value. FDS curve data analysis was carried out using the commercially available SPIP software (Image Metrology, Denmark) and A/S and XEI software (Park Systems, Korea).

### Immunohistochemistry and histology

All analysis was blinded to the identity and experimental condition of the animal/tissue. Mice were anesthetized by 300 µl of ketamine/xylazine cocktail, followed by trans-cardiac perfusion with 1× PBS at 7 weeks after injury. Brains were carefully removed, post-fixed in 4% paraformaldehyde for 2 days, and placed in 30% sucrose solution. Brains were then embedded in agarose and cross-sections were cut at room temperature at 40-μm thickness on a vibratome (Leica vt 1200S). Ten sections/mouse were randomly chosen for staining.

#### Immunohistochemistry

Free floating immunohistochemistry was done using tris-buffered saline+0.1% tween-20 (TBST) as the wash buffer and 10% donkey normal serum (cat. #ab7475, Abcam, USA) in PBS as the blocking buffer. A permeabilization step was added (prior to blocking and staining) using 0.1% triton and 0.6% hydrogen peroxide in 1× TBST. The following primary antibodies/reagents were used (diluted in blocking buffer): rabbit polyclonal anti-mCherry (1:1000, cat. #LS-C147181-30; Lifespan Biosciences, USA), rabbit polyclonal anti-parvalbumin (1:10,000; cat. #PV25; Swant Inc., Switzerland), biotinylated Wisteria Floribunda Lectin (1:1000, cat. #B-1355; Vector labs, USA), mouse monoclonal anti-GFP (anti-green fluorescent protein; 1:200, cat. #ab184601; Abcam, USA), and rabbit polyclonal anti-Aggrecan (1:500, cat. #AB1031; Millipore, USA). The following secondary antibodies were used (diluted in 1× TBST): Donkey anti-rabbit IgG (H+L) AlexaFluor 594 (1:500, cat. #711-585-152; Jackson Immunoresearch, USA), Donkey anti-rabbit IgG (H+L) AlexaFluor 488 (1:500, cat. #711-545-152; Jackson Immunoresearch, USA), Alexa Fluor® 555 streptavidin (1:500, cat. #S32355; ThermoFisher, USA), and Donkey anti-mouse IgG (H+L) AlexaFluor 488 (1:500, cat. #715-545-150; Jackson Immunoresearch, USA). Sections were counterstained with DAPI and mounted on slides using fluoromount aqueous mounting medium (cat. #F4680, Sigma, USA). Images were analyzed as a 2-μm-step *z*-stack of 20 slices (×20 objective magnification) using fluorescent imaging (Keyence BZ-X700, USA). Five sections per mouse were randomly chosen for analysis. WFA^+^ and PV^+^ cells were manually quantified in each *z*-stack using the “Count” tool and normalized to tissue area using Adobe Photoshop CS6. Matrix metalloproteinase 8 (MMP8) staining was quantified using the “Integrated Density” measure in Image J and normalized to tissue area (Bethesda, USA).

#### Histology

Analysis of overall matrix remodeling based on alterations in proteoglycan content was carried out using the multichromatic FAST (fast green, Alcian blue, Safranin O, and Tartrazine) staining method [18]. The staining sequence was as follows: distilled water—1 min, Alcian blue (strongly sulfated glycoproteins)—10 min, Safranin O (general detection of glycosaminoglycans)—2.5 min, 50% ethanol—1 min, Tartrazine (mucin-associated ECM)—10 s, Fast green (glycoprotein-rich structures)—5 min. Slides were mounted using DPX mounting medium (cat. #44581, Sigma, USA). Sections were imaged (×10 objective magnification) using brightfield microscopy (Keyence BZ-X700, USA). Five sections per mouse were randomly chosen for analysis. RGB values of each pixel of 40 images (1440 × 1920) were extracted. RGB values higher than (250, 250, 250) were omitted from the extracted values in order to exclude white background color from the analysis. The RGB values were then converted to HSV values, which better represents the spectrum of human color perception. The converted HSV values were binned into 256 different ranges. Each bin count was divided by the total count of all HSV colors in each picture to acquire the relative proportion of each binned color with respect to all binned colors. All analysis was done using Matlab 2016.

### Analysis of ECM proteins

All analysis was blinded to the identity and experimental condition of the animal/tissue. Mice were anesthetized (isoflurane) and sacrificed by decapitation at 7–8 weeks after injury. After the quick and careful removal from the skull, the hippocampus was extracted according to bregma coordinates and stored at −80 °C until use.

#### Two-color fluorescence western blotting

Western blot analysis was performed according to standard procedures. Briefly, after sodium dodecyl sulfate–polyacrylamide gel electrophoresis and blotting, proteins on the membranes were detected by overnight incubation at 4 °C with the primary antibody followed by incubation with an infrared dye (IRDye)-conjugated secondary antibody. The following primary antibodies were used: rabbit monoclonal anti-Hapln1 (hyaluronan and proteoglycan link protein 1, 1:1 000, cat. #ab181997; Abcam, USA), rabbit polyclonal anti-Aggrecan (1:500, cat. #AB1031; Millipore, USA), rabbit polyclonal anti-HS2 (anti-hyaluronan synthase 2, 1:1000, cat. #ab199794; Abcam, USA), mouse monoclonal anti-Neurocan (1:1000, cat. #ab26003; Abcam, USA), mouse monoclonal anti-Brevican (1:20, cat. #73-281; Neuromab, USA), rabbit polyclonal anti-Versican (1:500, cat. #AB1032; Millipore, USA), and mouse monoclonal anti-Chondroitin 4 Sulfate (1:1000, cat. #MAB2030; Millipore, USA). The following secondary antibodies were used: IRDye 800CW goat anti-mouse IgG (H+L) (1:20,000; cat. #926–32210; LI-COR Biosciences, USA) and IRDye 680RD goat anti-rabbit IgG (H+L) (1:20,000; cat. #925–68071; LI-COR Biosciences, USA). β-Actin was used as an internal control and was detected with the mouse monoclonal anti-β-actin antibody (1:5000, cat. #ab6276; Abcam, USA) followed by incubation with an IRDye 680CW goat anti-mouse IgG (H+L) (1:20,000; cat. #926–32220; LI-COR Biosciences, USA). The signals were detected using Odyssey (LI-COR Biosciences, USA) and quantified using the Image Studio software.

#### Enzyme-linked immunosorbent assay (ELISA)

Levels of MMP2, 3, 8, and 9 were measured by multiplex ELISA (Eve Technologies, Canada). Tissue inhibitor of metalloprotease 1 (TIMP1) and TIMP2 levels were measured using commercially available kits per the manufacturer’s instructions (cat. #ab196265, #ab100746, Abcam, USA)

### MMP8 downregulation

All analysis was blinded to the identity and experimental condition of the animal/tissue.

We manipulated MMP generation capacity in hippocampal cells using short hairpin RNA (shRNA) administration through lentiviral delivery. Lentiviral particles for mouse MMP8-specific shRNA (MMP8 Mission^®^ shRNA; 1 × 10^6^ TU/mL; pLKO.1 vector) or non-target shRNA control (Mission^®^ pLKO.1 puro non-target shRNA control) were purchased commercially (Sigma Aldrich) and used to downregulate MMP levels in the hippocampus. The sequence for MMP8-specific shRNA is: CCGGGCCTTGATGTACCCAAACTATCTCGAGATAGTTTGGGTACATCAAGGCTTTTTG. Mice received single intracranial injections of lentivirus into each of the left and right hemispheres 3 weeks after injury. The injection coordinates were AP = −2mm from bregma and ML = ± 1.6 mm from midline to deliver the lentivirus to the DG. The injection depth was 2.2 mm from the dura. A total of 1 µl was injected in each hippocampus at the rate of 0.1 µl/min. To visualize lentiviral spread, a pLKO.1-CMV-tGFP vector with a non-target SHC016 shRNA sequence (Sigma Aldrich, USA) was used as described above.

### Electrophysiology

All analyses were blinded to the identity and experimental condition of the animal/tissue.

Following deep anesthesia (isoflurane) and sacrifice, the brain was quickly removed into ice-cold artificial cerebrospinal fluid (ACSF) bubbled with 5% CO_2_/95% O_2_ continuously. The ACSF was constituted as (in mM) NaCl 131.0, KCl 2.5, KH_2_PO_4_ 1.2, CaCl_2_ 2.4, MgSO_4_ 1.3, NaHCO_3_ 26.0, and glucose 10.0 (pH 7.4). Right hippocampus was cut into slices (400 µm thick) with a tissue slicer (Stoelting Co., IL) and incubated in room temperature with ACSF oxygenated continuously for at least 1 h. Extracellular recording was made in a Haas chamber (Harvard Apparatus, USA) with submerged mode at room temperature. Slices were continuously perfused with ACSF bubbling with 5% CO_2_/95% O_2_ at flow rate about 1.5 ml/min with a peristaltic pump (Buchler, USA).

The recording electrode was made with borosilicate glass (Warner Instruments) and filled with regular ACSF (resistance = ~1–3MΩ). Biphasic current stimulating pulses (0.4 ms) were delivered with an interval of 10 s through concentric bipolar electrode (CBARC75, FHC). To record field population spikes in the dorsal DG, the recording electrode was placed at the lateral part of upper granular cell layer and stimulating electrode was placed immediately above hippocampal fissure to tease the bypassing perforant pathway fibers. Input/output curves were obtained for each slice with stimulus intensities ranging from threshold to 1.0 mA. Baseline was recorded with stimulus intensity evoking half of maximal response. High frequency stimulation consisted of 2 × 100 pulse trains (1 s, 100 Hz) in baseline stimulus intensity. Slices were recorded within 6 h after dissection. Data were acquired with an Axopatch 2B amplifier through Digitizer 1320A using ClampEx 10.4 (Molecular Devices, USA).

The amplitude and slope of population spikes were measured from the initial phase of negative wave with Clampfit10.4 (Molecular Devices, USA). Each data point was measured as an average of three consecutive traces. LTP following high frequency stimulation was plotted as a percentage of the baseline. Representative traces are the average of 6 consecutive stimulations in a 1-min period. Paired pulse ratio (PPR) was recorded with baseline stimulus intensity in 50 ms inter-pulse interval. PPR was calculated as a percentage of the difference between the two responses divided by the first one.

### Statistical analysis

All data are expressed as mean ± s.e.m. (standard error of mean). Analysis of repeated parametric measures was accomplished using a two-way analysis of variance (ANOVA) followed by Holm–Sidak post hoc test for multiple comparisons. When comparing three groups, a one-way ANOVA was used followed by Holm–Sidak post hoc test for multiple comparisons. For simple comparisons of two groups, two-tailed Student's *t* test was used. Welch’s correction was used when the assumption of equal variances was not met. Significance was set at *P* value <0.05 (Prism 5; GraphPad Software, USA). Sample size for each experimental end point is indicated in the figures.

## Results and Discussion

**Fig. 1:**
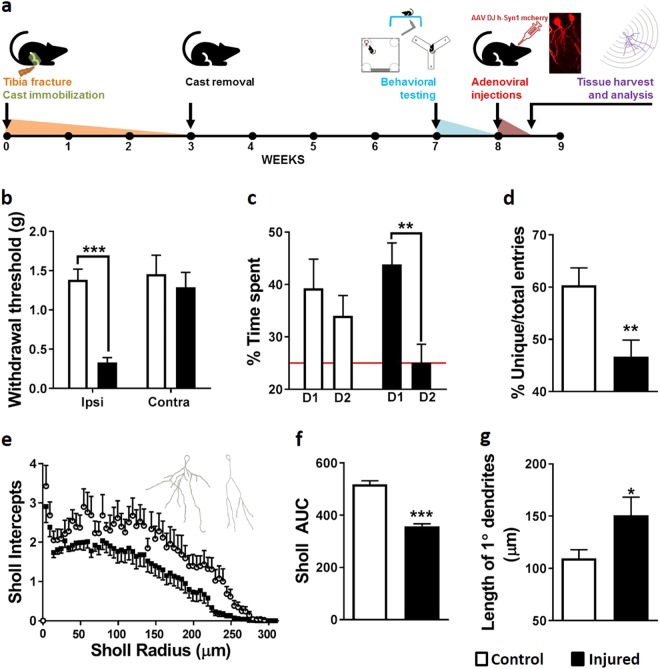
Alterations in hippocampal cytoarchitecture parallel behavioral signs of pain and memory dysfunction following peripheral injury. **a** Experimental timeline: Male C57BL/6 mice underwent tibia fracture and cast immobilization. All analysis was carried out during the 7–9-week period. **b** Seven weeks following peripheral injury, mice display a reduction in mechanical thresholds on the ipsilateral hindpaw (two-way ANOVA, post hoc Holm–Sidak test for multiple comparisons, *n* = 16–17 mice/group). **c** Injured mice are deficient in location memory recall 1 day following exposure to a female mouse (two-way ANOVA, post hoc Bonferroni test for multiple comparisons, *n* = 7–9 mice/group). **d** Similarly, they demonstrate deficits in working spatial memory in the Y maze as evidenced by the percentage of unique triad combination of consecutive arm entries (Student's *t* test, *n* = 8–9 mice/group). **e**–**g** AAV DJ-hSyn1 mCherry was stereotaxically injected into the hippocampus to fluorescently label a small percentage of neurons. Representative neuronal tracings are included in the inset (left = Control, right = Injured). Sholl analysis revealed decreased dendritic complexity following injury, in addition to increased length of primary dendrites. (Student's *t* test, *n* = 10–15 mice/group). Error bars are s.e.m. *P < 0.05; **P < 0.01; ***P < 0.005

We used a mouse model of CP due to tibia fracture to demonstrate, 7 weeks following injury (Fig. 1a), behavioral signs of tactile allodynia indicated by reduced mechanical thresholds on the injured hindpaw (Fig. 1b). Even though the tibia fracture itself had healed, at this chronic time point, injured mice display signs of pain as well as memory deficits in the location memory test (long-term memory, Fig. 1c) and the Y maze (working memory, Fig. 1d). We therefore examined hippocampal neuronal architecture by fluorescently labeling a subset of hippocampal neurons via adenoviral delivery (AAV DJ-hSyn1 mCherry). We focused on the hippocampal DG due to its role in adult neurogenesis and plasticity [19], particularly in CP paradigms [20]. Sholl analysis of dentate granular cells showed an overall decrease in dendritic complexity (Fig. 1e, f) that parallels increased dendritic length (Fig. 1g) in the injured group. These observations reflect hippocampal plasticity that persists long after the initial injury to the limb, and considering that pain encompasses affective-motivational and cognitive-evaluative dimensions, these signs of hippocampal plasticity not only parallel the memory deficits observed in CP but also further bolster the role of the hippocampus in pain processing [21]. These findings complement previous reports of pain-related structural and functional changes in the hippocampus: compared to control subjects, patients with CP demonstrated increased hippocampal gray volume [8] and increased hippocampal–prefrontal cortex connectivity [9, 10]. Similarly, changes in hippocampal volume were observed in preclinical models of pain [11]. Finally, we have reported biochemical changes in the form of decreased hippocampal levels of synaptophysin and brain-derived neurotrophic factor in mice with peripheral injury [7].

**Fig. 2:**
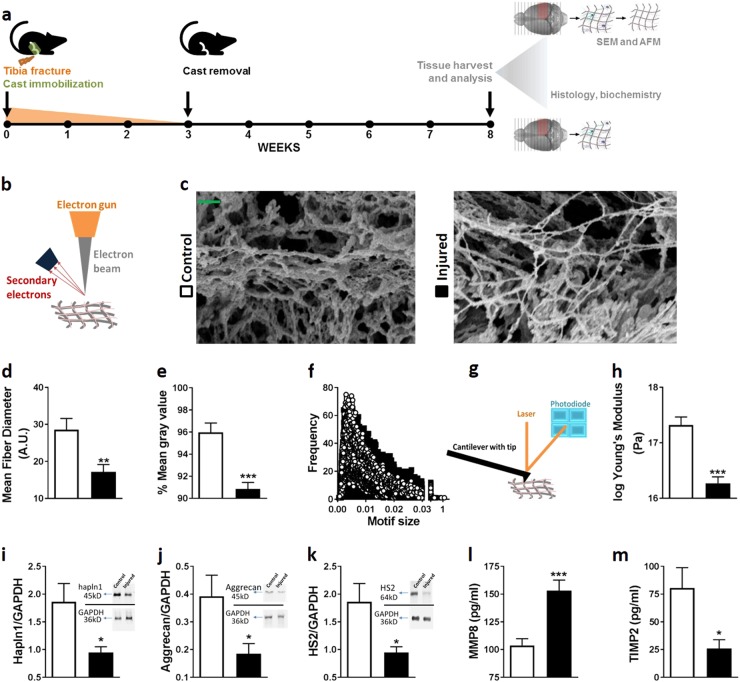
Peripheral injury is accompanied by biophysical and biochemical alterations in the hippocampal extracellular matrix. **a** Experimental timeline: Male C57BL/6 mice underwent tibia fracture and cast immobilization. Seven weeks following injury, brains were decellularized for scanning electron microscopy (SEM) and atomic force microscopy (AFM) analysis or left intact for histological and biochemical analyses. **b**–**f** Surface scanning electron micrographs demonstrated changes in ECM microarchitecture in the form of altered geometrical micromotifs, and reduced mean fiber diameter as well as a reduction in mean gray values. Scale bar = 400 nm. (Student’s *t* test, *n* = 5 mice/group). **g**, **h** The examination of ECM stiffness using contact-mode AFM revealed decreased ECM rigidity in the hippocampi of injured mice (Student’s *t* test, *n* = 22 measurements each taken from 5 mice/group). **i**–**m** Injury is associated with decreased levels of hapln1, aggrecan, and HS2, in addition to decreased levels of MMP8 and increased levels of TIMP2 (Student’s *t* test, *n* = 8–12 mice/group). Error bars are s.e.m. *P < 0.05; **P < 0.01; ***P < 0.005

Since the advent of the neuron doctrine [22], the majority of brain plasticity studies have focused on neurons, with a more recent interest in glia. However, it is well known that the ECM in the CNS is crucial in the regulation and stabilization of plasticity, including synaptic activity, both during development and in adulthood [23]. Thus, a flexible response by the ECM is necessary to permit and/or facilitate cellular alterations. To understand whether global ECM changes in the hippocampus occur in response to pain, we isolated the ECM by decellularizing the dissected hippocampi (Fig. 2a), and measured structural changes using surface SEM and contact-mode AFM. Injured animals displayed altered ECM microarchitecture in the form of thinner and less abundant ECM fiber as well as alterations in geometrical motifs compared to controls (Fig. 2b–f). Furthermore, injury was associated with reduced stiffness of the hippocampal ECM, evidenced from the change in Young’s modulus (Fig. 2g, h) measured by force–distance AFM indentation measurements. Our findings of decreased ECM stiffness and decreased dendritic complexity complement studies where rigid ECM substrates were shown to favor increased dendritic numbers and branching while a softer ECM was linked to fewer and longer neurites and an increased number of synapses [24, 25].

Our biophysical measures support the hypothesis that, in the adult brain, peripheral injury is linked to significant extracellular alterations of the ECM at the structural level. What, then, are the individual ECM components involved? The brain ECM that occupies ~20% of adult brain volume is mainly composed of proteoglycan and hyaluronan components [26] and is synthesized by neuronal, glial, and endothelial cells. Based on our AFM differences in structural rigidity, we focused on structural—as opposed to matricellular—ECM components. We first stained the tissue for proteoglycans based on their electric charge using the multichromatic FAST (see Methods). Our results show qualitative differences between the groups, where safranin O staining appears to be reduced after injury, indicating diminishing glycosaminoglycan content (Supplementary Fig. 1). Next, we quantified biochemically both the various components of the ECM and ECM-modulating enzymes. We found decreased levels of hapln1 and aggrecan (Fig. 2i, j), two key components of the specialized network of ECM known as the perineuronal net (PNN), in addition to decreased levels of HS2 (Fig. 2k), an enzyme that synthesizes hyaluronic acid, the backbone of the ECM. This profile is similar to that observed in the hippocampus during status epilepticus, where enhanced neuronal activity is associated with decreased aggrecan, hapln1, and HS3 [27]. Additionally, we show increased levels of MMP8 (Fig. 2l), an aggrecan-degrading metalloprotease, and decreased levels of TIMP2 (Fig. 2m). MMPs are well known as plasticity stimulators and mediators of synaptic physiology and neurite outgrowth. Furthermore, MM8 inhibition protects against systemic inflammation [28] and its upregulation in CSF can serve as a biomarker of spinal cord injury [29]. More recently, MMP8 was shown to act as a neuroinflammatory mediator in activated microglia [30] and to play a critical role in brain damage following ischemic injury [31]. TIMP2 is an endogenous protein that reversibly inhibits a wide range of MMPs—including MMP8—in a 1:1 stoichiometric manner [32], and a disturbed balance between TIMPs, MMPs, and ECM proteins is often seen in various neurodegenerative pathologies [33, 34]. More specifically, a recent study has shown that TIMP2 improves hippocampal cognition in aged mice [35]. No changes were observed in the abundance of neurocan, brevican, versican, chondroitin 4 sulfate, MMP2, MMP3, pro-MMP9, and TIMP1 (Supplementary Fig. 2).

**Fig. 3:**
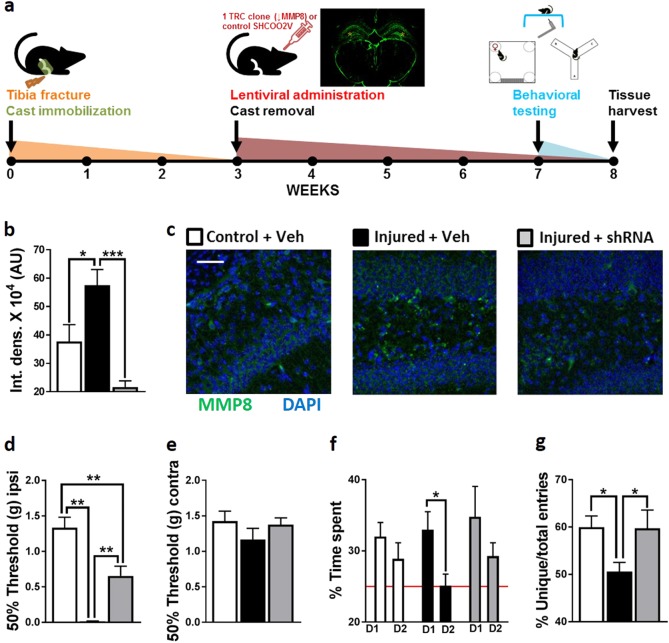
**a** Experimental timeline: Male C57BL/6 mice underwent tibia fracture and cast immobilization. Three weeks following injury, ↓MMP8 or control shRNA was injected in bilateral hippocampi (inset shows fluorescent GFP labeling of lentiviral spread, yellow asterisks indicate the site of injection). Behavioral and immunohistochemical analyses were carried out 7–8 weeks after injury. **b**, **c** Upregulated MMP8 levels in injured mice were normalized following shRNA treatment (one-way ANOVA, post hoc Holm–Sidak test for multiple comparisons, *n* = 10–12 mice/group). Scale bar = 100 µm. **d**–**g** MMP8 downregulation ameliorates signs of mechanical allodynia in the ipsilateral hindpaw (no differences were seen in the contralateral hindpaw) as well as memory dysfunction (female location memory and Y maze) following injury (one-way ANOVA (**d**, **f**) or two-way ANOVA (**e**), post hoc Holm–Sidak test for multiple comparisons, *n* = 9–16 mice/group). Error bars are s.e.m. *P < 0.05; ***P < 0.005

Since our data suggest an altered state of homeostatic ECM plasticity after peripheral injury, we next tested whether the restoration/prevention of ECM dysregulation could result in the amelioration of the nociceptive and cognitive changes observed in CP. Our target of choice was MMP8 instead of TIMP2 due to specificity, since it was the only MMP that was upregulated after injury and since it can degrade hapln1 and aggrecan, two ECM components that were downregulated in our model. To assess the functional role of MMP8 in contributing to the broader CP phenotype, we downregulated MMP8 levels in bilateral hippocampi using lentiviral-delivered shRNA (Fig. 3a–c). This decrease in hippocampal MMP8 resulted in improvements in tactile allodynia in the ipsilateral but not in the contralateral hindpaw (Fig. 3d, e) as well as improvements in long-term memory in the female location memory assay (Fig. 3f) and working memory in the Y maze (Fig. 3g). To our knowledge, this is the first report linking MMP8 to hippocampal and behavioral plasticity.

**Fig. 4:**
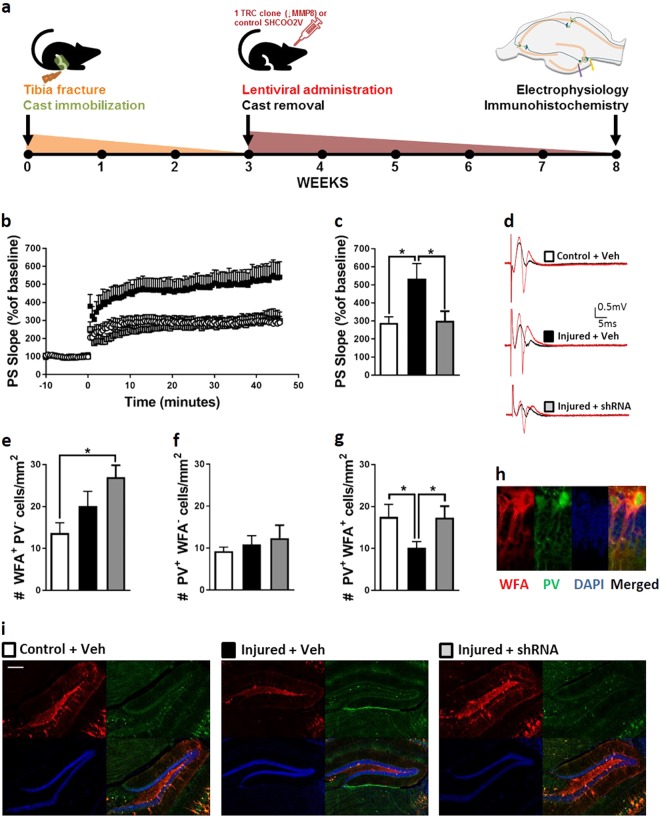
The stabilization of hippocampal inhibitory interneurons by the perineuronal net is disrupted following peripheral injury. **a** Experimental timeline: Male C57BL/6 mice underwent tibia fracture and cast immobilization. Three weeks following injury, ↓MMP8 or control shRNA lentiviral vector was injected bilaterally in hippocampal formation. Electrophysiological and immunohistochemical analyses were carried out 8 weeks after injury. **b**–**d** Injured mice demonstrated increased field EPSP recorded from the DG, which was normalized following MMP8 downregulation (one-way ANOVA, post hoc Holm–Sidak test for multiple comparisons, *n* = 13 slices from 4 mice/group). **e**–**i** Injury is not linked to a loss of non-parvalbuminergic cells surrounded by a perineuronal net (WFA^+^ PV^−^) or a decrease in the numbers of parvalbumin-positive interneurons that lack a perineuronal net (WFA^−^ PV^+^). However, immunohistochemical examination showed that injured mice have decreased numbers of inhibitory interneurons that are surrounded by a perineuronal net (WFA^+^ PV^+^) in the DG, suggesting a loss of inhibitory drive in these animals. MMP8 downregulation results in the normalization of WFA^+^ PV^+^ cell counts (one-way ANOVA, post hoc Holm–Sidak test for multiple comparisons, *n* = 6–10 mice/group). Scale bar = 200 µm. Error bars are s.e.m. *P < 0.05

Having demonstrated both neuronal and extracellular alterations in the hippocampus in CP, we aimed to delineate the mechanistic link between the two. Electrophysiological analysis of LTP in the dorsal DG showed an aberrant increase in field excitatory postsynaptic potential in CP mice that was absent in the shRNA-treated mice (Fig. 4b–d). These data complement findings from animal studies where chronic visceral pain was shown to be linked to enhanced hippocampal LTP [36] as well as human studies where a widespread increase in hippocampal connectivity was observed in patients with chronic low back pain [10]. No changes in input/output or PPR measures were noted, suggesting that injury is not linked to decreased synaptic transmission efficiency or decreased probability of neurotransmitter release (Supplementary Fig. 3).

Unlike LTP in immature brains, the post-injury aberrant LTP levels in an adult hippocampal granular cell layer following high frequency stimulation implies a need for reduced GABA-ergic inhibition [37]. Indeed, recent studies have shown a role for GABA-ergic signaling in modulating synaptic plasticity in the adult brain [38] as well as evidence for parvalbumin-positive interneurons regulating hippocampal LTP [39]. Since many hippocampal inhibitory interneurons are surrounded by a PNN, and since GABA-ergic transmission and PNN alterations are classically viewed as “plasticity brakes” [40], we therefore hypothesized that a possible mechanism by which ECM can alter neuronal physiology is through the stabilization of inhibitory GABA-ergic interneurons in the hippocampus through the PNN that surrounds their cell bodies and proximal neurites. Chondroitin sulfate proteoglycans, including aggrecans, are main constituents of the PNN (Supplementary Fig. 4) and generally act to restrict adult neuroplasticity [41]. Our immunohistochemical analysis of parvalbumin-positive inhibitory interneurons that are surrounded and stabilized by a PNN (evidenced by wisteria floribunda lectin staining) demonstrated a decline in the number of stable inhibitory interneurons in the DG (Fig. 4e-i) but not in the CA regions (Supplementary Fig 5). This decrease was normalized in the shRNA-treated group. No changes in the number of parvalbumin-positive inhibitory interneurons that are not surrounded by a PNN were observed in the DG or CA regions after injury (Fig. 4f, Supplementary Fig. 5b).

Despite mounting evidence for the role of hippocampal remodeling after an injury to the periphery and CP, the adult hippocampal ECM is often erroneously thought to be static. Our results show that the sustained anatomical, physiological, and biochemical dysregulation of the hippocampal DG ECM after peripheral injury supports pain as well as cognitive deficits characteristic of pain patients. Therapies directed at controlling maladaptive ECM plasticity may constitute a novel approach to controlling CP and its undesirable sequelae, going beyond examining nociceptive pathways themselves, and addressing structural factors supporting the broader pain experience.

## Electronic supplementary material

Figure S1: Hippocampal proteoglycan content is altered in injured mice

Figure S2: Biochemical analysis of various ECM components and enzymes

Figure S3: Peripheral injury is not linked to diminished synaptic transmission or neurotransmitter release probability in the hippocampus

Figure S4: Aggrecan co-localizes with WFA

Figure S5: Injury is not linked with alterations in the number of stable inhibitory interneurons in the hippocampal CA region
