# Supplementary material for: The hippocampal extracellular matrix regulates pain and memory after injury
Source: Mol Psychiatry. 2018 Sep 26;23(12):2302–13. doi: 10.1038/s41380-018-0209-z (PMC6294737; doi:10.1038/s41380-018-0209-z)
Supplement: Supplementary file 1 — Figure S1: Hippocampal proteoglycan content is altered in injured mice [file 41380_2018_209_MOESM1_ESM.pdf]

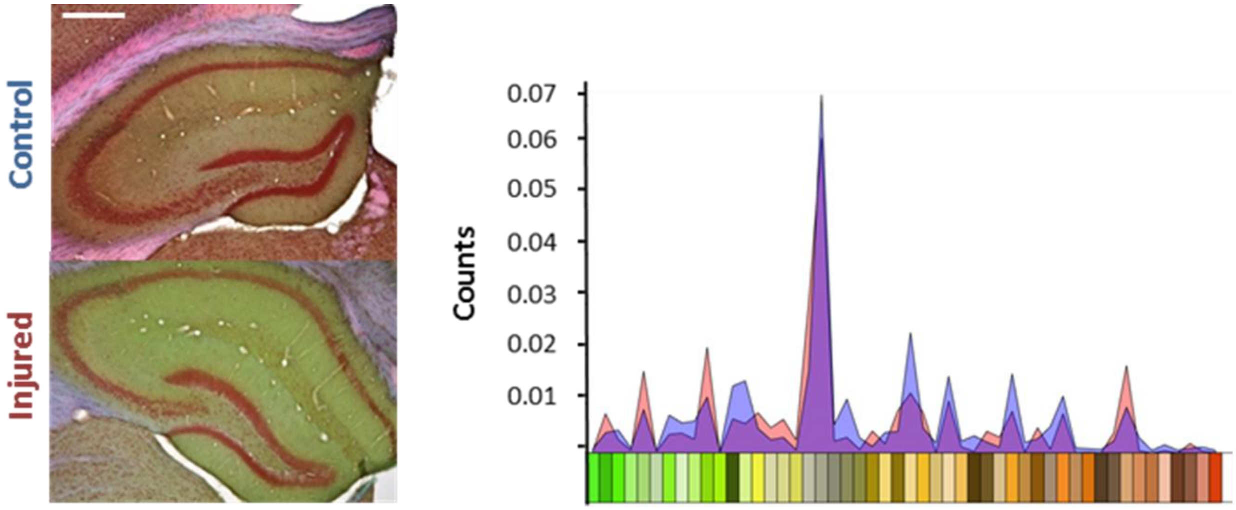

Figure S1: Hippocampal proteoglycan content is altered in injured mice. Histological analysis of proteoglycan content shows significant changes in color composition in injured mice (Student's t-test, n=5-8mice/group). Scale bar=400 µm.
