# Supplementary material for: The hippocampal extracellular matrix regulates pain and memory after injury
Source: Mol Psychiatry. 2018 Sep 26;23(12):2302–13. doi: 10.1038/s41380-018-0209-z (PMC6294737; doi:10.1038/s41380-018-0209-z)
Supplement: Supplementary file 2 — Figure S2: Biochemical analysis of various ECM components and enzymes [file 41380_2018_209_MOESM2_ESM.pdf]

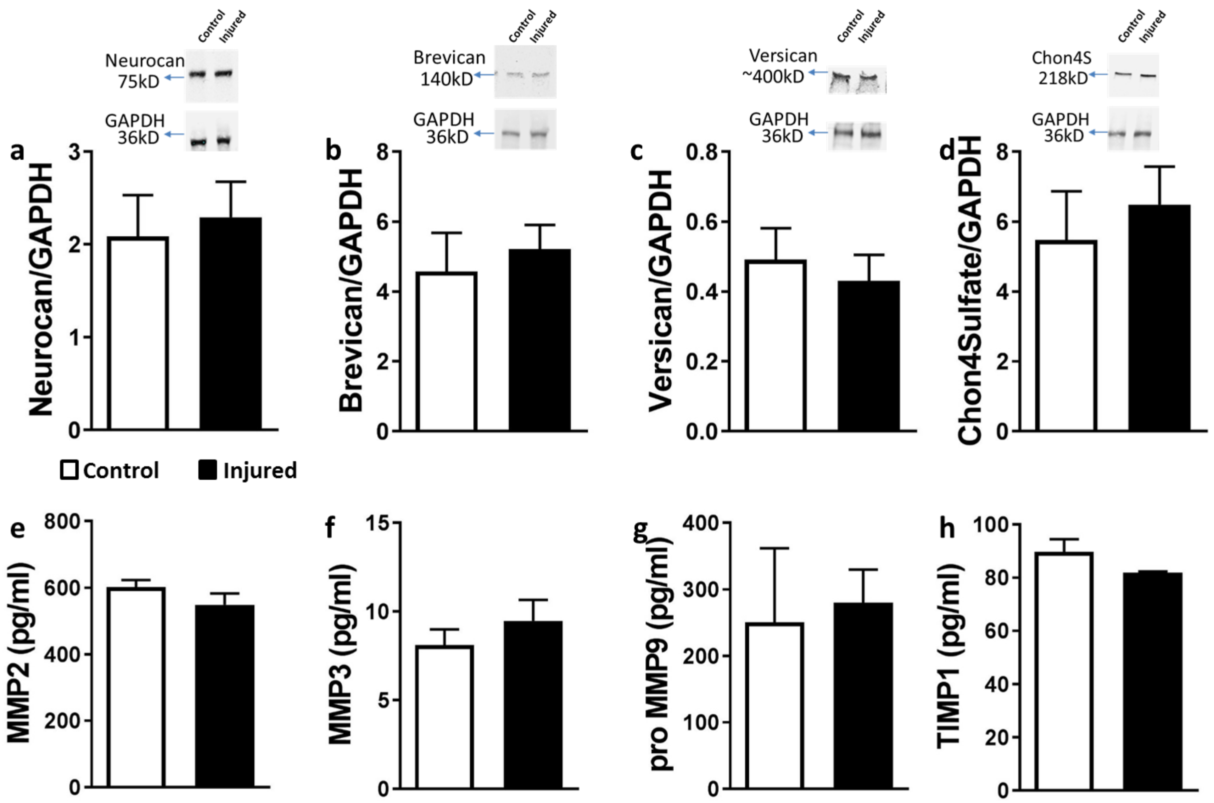

Figure S2: **Biochemical analysis of various ECM components and enzymes.** (**a-h**) Biochemical analysis of hippocampi from 7-week old control and injured mice shows no differences in the levels of neurocan, brevican, versican, chondroitin 4 sulfate, MMP2, MMP3, pro-MMP9, and TIMP1 (Student t-test, n=4-8 mice/group). Error bars are s.e.m.
