# Supplementary material for: The hippocampal extracellular matrix regulates pain and memory after injury
Source: Mol Psychiatry. 2018 Sep 26;23(12):2302–13. doi: 10.1038/s41380-018-0209-z (PMC6294737; doi:10.1038/s41380-018-0209-z)
Supplement: Supplementary file 3 — Figure S3: Peripheral injury is not linked to diminished synaptic transmission or neurotransmitter release probability in the hippocampus [file 41380_2018_209_MOESM3_ESM.pdf]

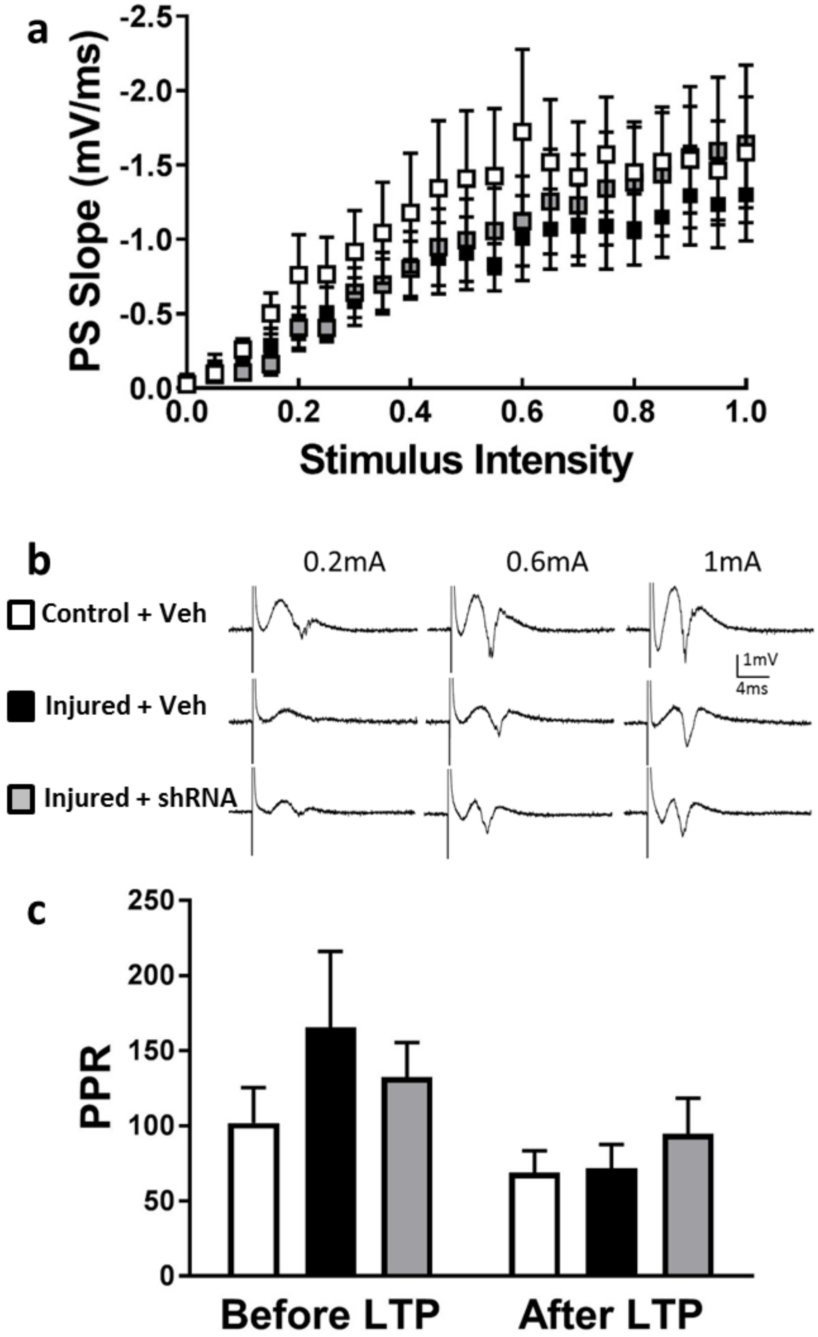

Figure S3: **Peripheral injury is not linked to diminished synaptic transmission or neurotransmitter release probability in the hippocampus.** (**a, b**) Seven weeks post injury, control and injured groups do not differ in their dentate gyrus input/output curves, suggesting that the efficiency of synaptic transmission is comparable between the 2 groups. MMP8 downregulation in the injured group was not associated with any change (2-way ANOVA, n=13/group). (**c**) Similarly, no differences in EPSP paired-pulse ratio (PPR; 50 ms interval) were observed among the 3 groups, implying no changes in the release probability of neurotransmitters after injury and/or treatment (2-way ANOVA, n=13/group). Error bars are s.e.m.
