# Supplementary material for: The hippocampal extracellular matrix regulates pain and memory after injury
Source: Mol Psychiatry. 2018 Sep 26;23(12):2302–13. doi: 10.1038/s41380-018-0209-z (PMC6294737; doi:10.1038/s41380-018-0209-z)
Supplement: Supplementary file 4 — Figure S4: Aggrecan co-localizes with WFA [file 41380_2018_209_MOESM4_ESM.pdf]

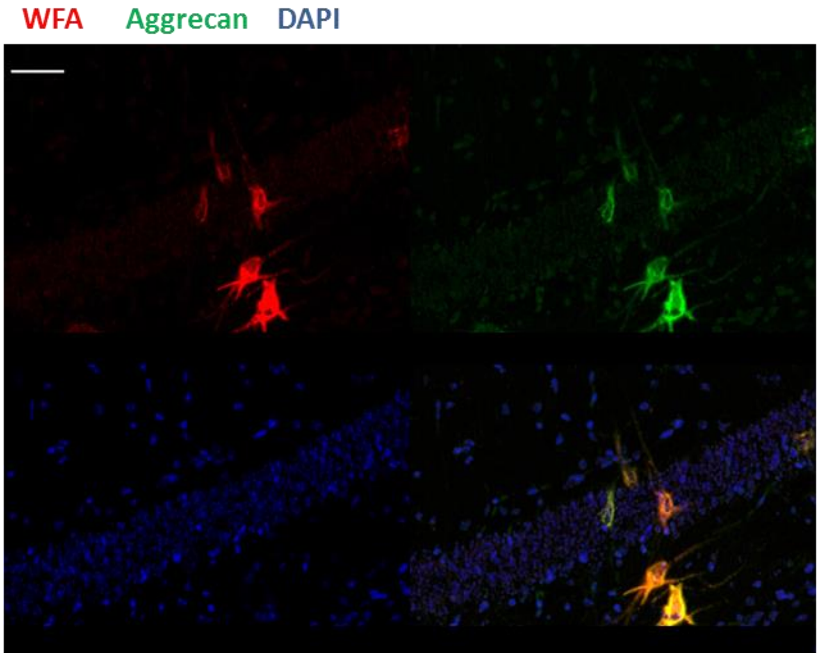

Figure S4: **Aggrecan co-localizes with WFA**. Aggrecan is a key component of the specialized extracellular matrix known as the perineuronal net (WFA stain). Scale bar=50µm.
