# Supplementary material for: The hippocampal extracellular matrix regulates pain and memory after injury
Source: Mol Psychiatry. 2018 Sep 26;23(12):2302–13. doi: 10.1038/s41380-018-0209-z (PMC6294737; doi:10.1038/s41380-018-0209-z)
Supplement: Supplementary file 5 — Figure S5: Injury is not linked with alterations in the number of stable inhibitory interneurons in the hippocampal CA region [file 41380_2018_209_MOESM5_ESM.pdf]

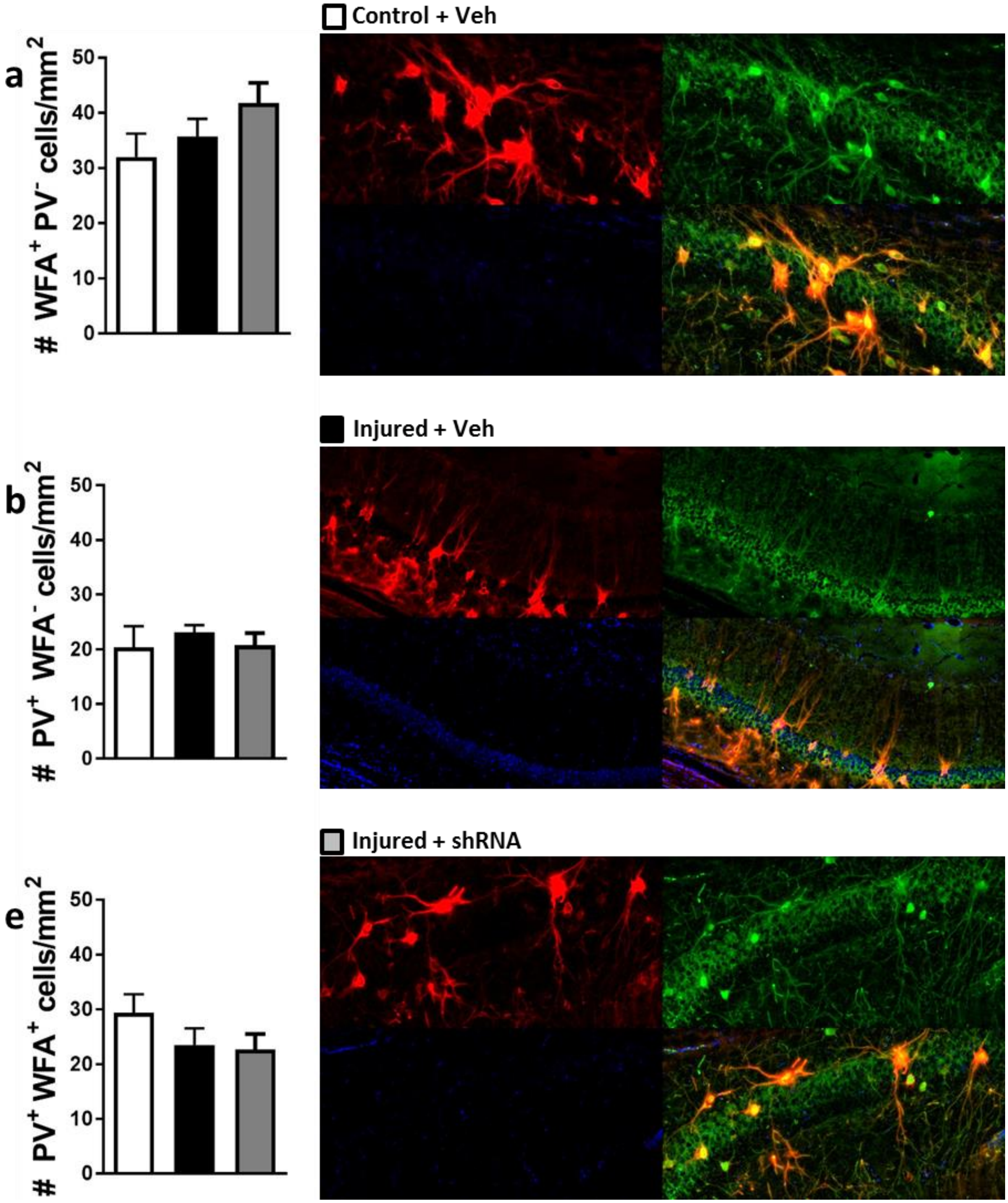

Figure S5: **Injury is not linked with alterations in the number of stable inhibitory interneurons in the hippocampal CA region.** (**a-c**) Injury is not linked to changes in the number of WFA$^+$ PV$^-$, WFA$^-$ PV$^+$, or WFA$^+$ PV$^+$ cells in the CA region of the hippocampus (One-way ANOVA, n=6-8 mice/group). No significant effects of MMP8 downregulation (shRNA treatment) were observed. Scale bar=200µm. Error bars are s.e.m.
